# The role of non-coding RNAs in the regulation of stem cells and progenitors in the normal mammary gland and in breast tumors

**DOI:** 10.3389/fgene.2015.00072

**Published:** 2015-02-27

**Authors:** Chiara Tordonato, Pier Paolo Di Fiore, Francesco Nicassio

**Affiliations:** ^1^Department of Experimental Oncology, European Institute of Oncology, MilanItaly; ^2^Fondazione Istituto FIRC di Oncologia Molecolare, MilanItaly; ^3^Dipartimento di Scienze della Salute, Università degli Studi di Milano, MilanItaly; ^4^Center for Genomic Science of IIT@SEMM, Istituto Italiano di Tecnologia, MilanItaly

**Keywords:** non-coding RNA, miRNA, lncRNA, stem cell, cancer stem cell, mammary gland, breast cancer

## Abstract

The outlook on stem cell (SC) biology is shifting from a rigid hierarchical to a more flexible model in which the identity and the behavior of adult SCs, far from being fixed, are determined by the dynamic integration of cell autonomous and non-autonomous mechanisms. Within this framework, the recent discovery of thousands of non-coding RNAs (ncRNAs) with regulatory function is redefining the landscape of transcriptome regulation, highlighting the interplay of epigenetic, transcriptional, and post-transcriptional mechanisms in the specification of cell fate and in the regulation of developmental processes. Furthermore, the expression of ncRNAs is often tissue- or even cell type-specific, emphasizing their involvement in defining space, time and developmental stages in gene regulation. Such a role of ncRNAs has been investigated in embryonic and induced pluripotent SCs, and in numerous types of adult SCs and progenitors, including those of the breast, which will be the topic of this review. We will focus on ncRNAs with an important role in breast cancer, in particular in mammary cancer SCs and progenitors, and highlight the ncRNA-based circuitries whose subversion alters a number of the epigenetic, transcriptional, and post-transcriptional events that control “stemness” in the physiological setting.

## BIOGENESIS AND FUNCTIONS OF miRNAs AND lncRNAs

The “non-coding revolution” has completely shifted our view of gene expression programs, which have historically been based on the assumption that only protein coding genes could specify cellular functions. Recent research has highlighted the existence of numerous species of non-coding RNAs (ncRNAs) and provided compelling evidence of a significant regulatory role of these molecules, impacting both on physiology and disease (Ambros, 2004; Bushati and Cohen, 2007; Gangaraju and Lin, 2009; Mercer et al., 2009; Ponting et al., 2009; Wilusz et al., 2009; Berezikov, 2011; Lujambio and Lowe, 2012). Indeed, the number of ncRNAs per genome correlates far better with organism complexity than the number of coding genes, suggesting that RNA-based regulatory mechanisms are critical in the evolution of developmental complexity (Taft et al., 2007).

Regulatory ncRNAs can be divided into two classes based on their length: small and long ncRNAs (lncRNAs). Small ncRNAs comprise the short (<200 nt) RNA species, such as small-interfering RNAs (siRNAs, 19–23 nt), piwi-interacting RNAs (piRNAs, 26–30 nt), and microRNAs (miRNAs, 18–25 nt). lncRNAs comprise different types of transcripts, 100s to 1000s of nucleotides long, which are usually classified according to their genomic localization relative to the protein coding genes: sense, antisense, intronic, and intergenic ncRNAs.

### miRNAs: BIOGENESIS AND FUNCTIONS

Since the discovery in 1993 of the first small regulatory RNAs, lin-4 and let-7, which control the timing of *C. Elegans* larval development (Lee et al., 1993; Reinhart et al., 2000), thousands of different miRNAs have been identified in different organisms, including plants, animals and viruses (Lagos-Quintana et al., 2001). According to the last release of the miRNA database (***miRBase***  – release 21) 1881 precursor and 2588 mature miRNAs exist in the human genome (Kozomara and Griffiths-Jones, 2014).

**FIGURE 1:**
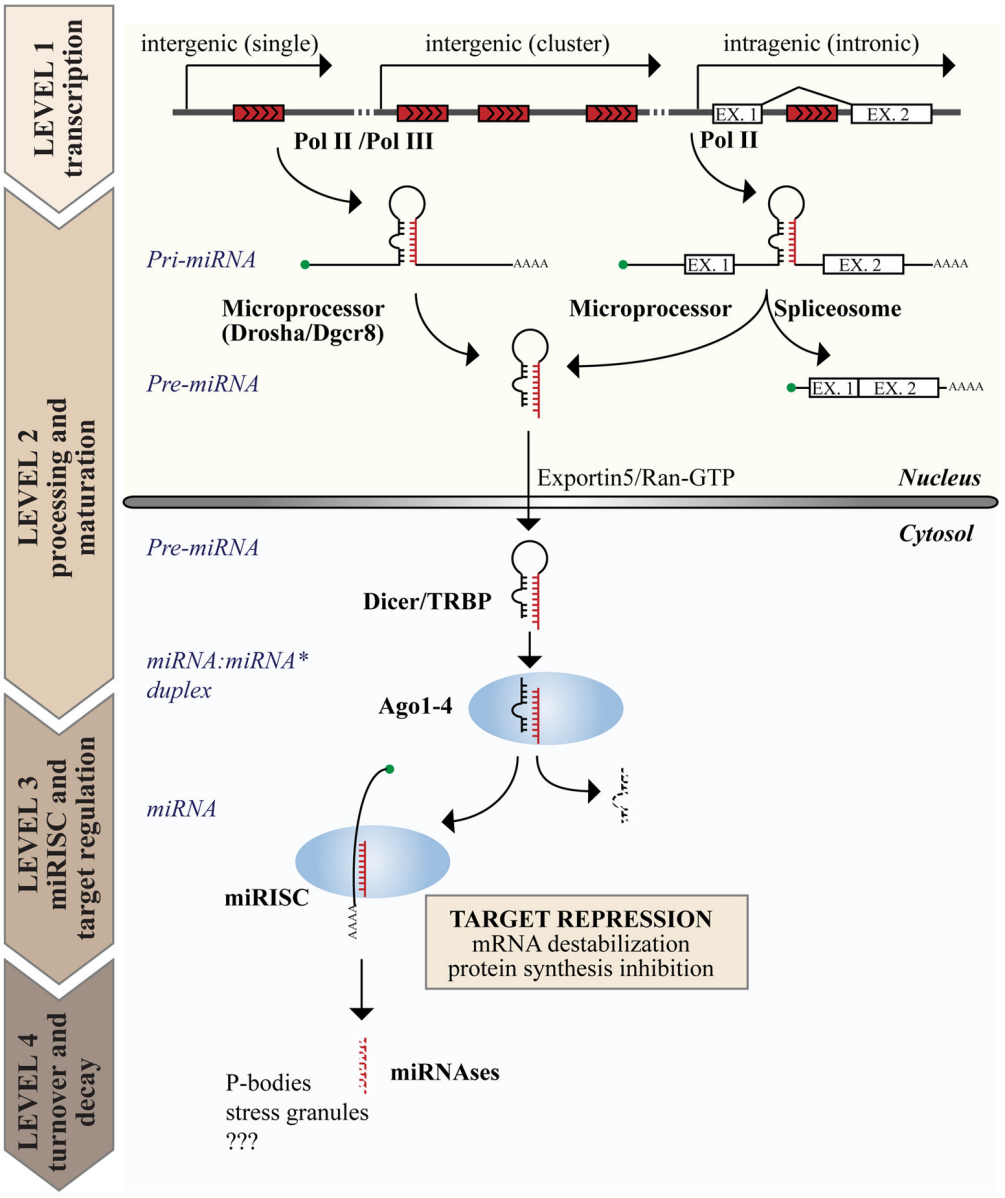
**Genome organization and biogenesis of microRNAs (miRNAs).** miRNA genes are interspersed in the genome with various possible locations as depicted on top. The figure summarizes the steps of the canonical miRNA biogenetic pathway, including the production of a primary transcript (pri-miRNA) by RNA polymerase II or III (Pol II or III), nuclear and cytoplasmic processing, loading into the miRISC complex, and the degradation of mature miRNAs. The function of miRNAs is exerted in the cytosol at the level of the miRISC, where miRNAs induce target gene repression by various mechanisms, including inhibition of protein synthesis and mRNA destabilization.

The biogenesis of miRNAs is a multistep process (reviewed in Ha and Kim, 2014 and summarized in **Figure 1**). The canonical pathway consists of at least four steps: transcription, nuclear, and cytoplasmic processing, loading into RNA-induced silencing complex (RISC) and decay. Transcription of miRNAs usually involves RNA polymerase II (Pol II), meaning that miRNA genes share the transcriptional machinery of protein coding genes, including transcription factors, enhancers, and epigenetic regulation. The genomic organization of miRNA genes also influences their transcription. Intragenic miRNAs, which constitute ∼40–45% of all human miRNAs, are co-transcribed with their “host gene” and, thus, share the same transcriptional regulation. Conversely, intergenic miRNAs form independent transcriptional units (Garzon et al., 2009). Occasionally, multiple miRNAs are organized in a single transcriptional unit, named “miRNA cluster” (Lee et al., 2002).

The expression of miRNAs begins with a long primary transcript, called the primary miRNA (or “pri-miRNA”), which contains a stem-loop region for each encoded miRNA and can range in size from hundreds of nucleotides to 10s of kilobases. Two processing events are required to generate the mature miRNA duplex, each generating an RNA molecule with a 5^′^ phosphate and a ∼2 nt 3^′^ overhang (Han et al., 2006). The first occurs in the nucleus and is mediated by the Microprocessor complex, composed of the RNase III endonuclease DROSHA and the DiGeorge syndrome Critical Region 8 (DGCR8) protein, which recognizes dsRNA–ssRNA junction and directs the cleavage site 11 nt away (Denli et al., 2004; Gregory et al., 2004). This cleavage event produces an intermediate hairpin precursor molecule of ∼65–100 nt, called the “pre-miRNA,” which is translocated from the nucleus to the cytoplasm by Exportin 5 and RAN–GTP (Lund et al., 2004). The second processing event occurs in the cytosol and is mediated by the RNase III endonuclease DICER in complex with the TAR RNA Binding Protein (TRBP; Bernstein et al., 2001). This last cleavage event generates the mature miRNA:miRNA^∗^ duplex (18–24 nt in length), which is immediately incorporated into the RISC, composed of Argonaute (AGO) proteins (Hammond et al., 2001; Mourelatos et al., 2002). However, only one strand of the duplex is retained in the RISC (the “guide” miRNA), while the other is discarded and degraded, resulting in a strong bias for guide strands in the miRNA pool (Khvorova et al., 2003; Schwarz et al., 2003). This miRNA maturation process is mainly regulated at the level of nuclear processing, and sequence determinants and auxiliary factors also contribute to its regulation (Auyeung et al., 2013; Mori et al., 2014).

There is evidence that a small proportion of miRNAs (<1%) are produced by non-canonical mechanisms, including: Drosha-independent mechanisms (as in the case of “mirtrons”), in which mRNA splicing produces a small RNA hairpin thereby bypassing Drosha processing (Berezikov et al., 2007); and Dicer-independent mechanisms (as in the case of miR-451), in which a short stem-loop is not loaded onto Dicer but directly processed by Ago2 (Yang et al., 2010).

Once loaded into the AGO-based complexes, miRNAs appear as rather stable molecules with long half lives (greater than 24 h); however, scattered reports suggest that miRNAs could also undergo a rapid and regulated decay (Krol et al., 2010; Rissland et al., 2011; Ruegger and Grosshans, 2012). Although miRNA biogenesis has been studied for 20 years, the mechanisms of miRNA degradation are largely obscure, in particular in higher organisms. At least two possible mechanisms have been proposed. The first involves the enzymatic activity of specific nucleases (“miRNases”), such as the plant SNDs (Wu and Belasco, 2008) and the worm 5^′^-to-3^′^ exoribonucleases XRN1 and XRN2 (Chatterjee and Grosshans, 2009). The second is a target-dependent mechanism, in which miRNA turnover is mediated by the interaction with mRNA targets that promote miRNA unloading from AGO and degradation (Baccarini et al., 2011; Ruegger and Grosshans, 2012; De et al., 2013).

Typically, miRNAs exert specific biological actions by interfering with a key regulator (e.g., a transcription factor) responsible for a defined phenotype (the “hub target” mechanism) or through the coordinated action on multiple target genes that belong to the same pathway (the “multiple targets” mechanism; Hausser and Zavolan, 2014). The interaction between miRNAs and their mRNA targets occurs in the cytosol at the level of the RISC and is directed by Watson–Crick base-pairing with the “miRNA Responsive Element” (MRE), usually located in the 3^′^-Untranslated Region (3^′^-UTR) of the target mRNA (Bartel, 2009). The critical determinant of miRNA specificity is a region located at the 5^′^ end of the miRNA in positions 2–8, called the “seed” sequence, which defines the target specificity of any mature miRNA. As the extent of miRNA:mRNA interaction is limited, 100s or even thousands of different genes are potential miRNA targets. These targets can be partially inferred with prediction algorithms that search in the 3^′^UTR of protein coding genes for the presence of MREs (i.e., miRanda, Targetscan, Pictar, DIANA microT, and RNAhybrid; Thomas et al., 2010). However, none of these algorithms are completely accurate, especially as some miRNA targets rely on “seedless” interactions.

Target down-modulation occurs through multiple mechanisms, including translational repression (inhibition of cap recognition or 60S recruitment, ribosome drop-off, and increase of termination efficiency) and/or mRNA destabilization (deadenylation or decapping). Rarely, in cases of perfect or near-perfect complementarity, miRNAs can function as siRNAs, inducing mRNA degradation (Carthew and Sontheimer, 2009).

### lncRNAs: BIOGENESIS AND FUNCTIONS

**FIGURE 2:**
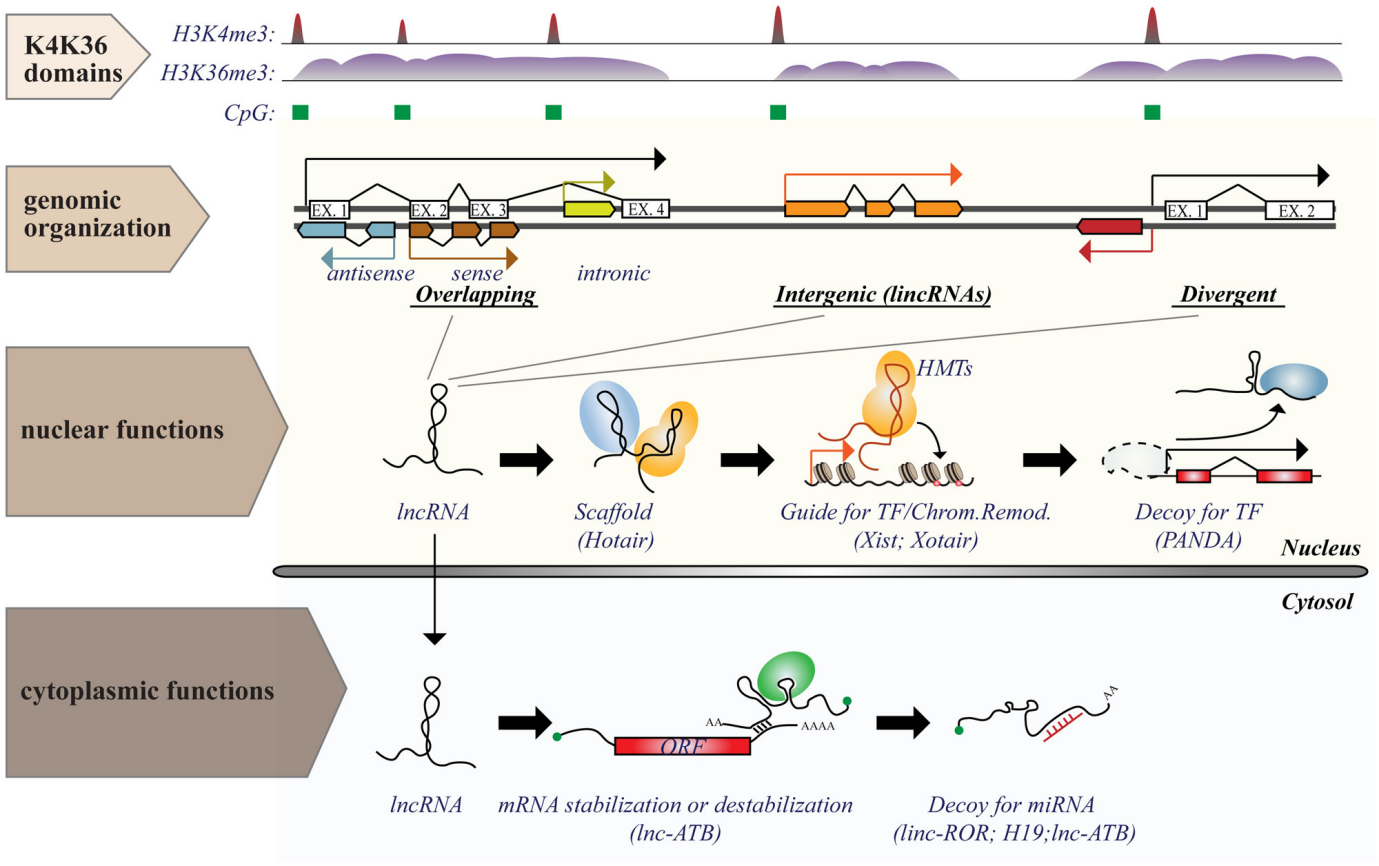
**Genomic organization and functions of long ncRNAs (lncRNAs).** lncRNA genes are interspersed in the genome in various possible locations in relation to protein coding transcripts, such as (i) overlapping; (ii) intergenic; or (iii) divergent transcripts. Transcription of lncRNAs follows the same rules as for protein coding genes and is executed by RNA Pol II. Genomic features associated with transcription [such as CpG island (green boxes) or histone marks (histone H3 “K4K36 domains”)] provide a useful strategy to identify expressed lncRNAs. Functions of lncRNAs are executed by multiple modes of action and can occur both in the nucleus and in the cytosol. The figure shows some examples of nuclear or cytoplasmic functions of some known lncRNAs.

Long ncRNAs consist of a heterogeneous class of ncRNAs, operationally defined as any RNA molecule with low coding potential and a size greater than 200 nt: a cut-off arbitrarily based on RNA purification protocols (Kapranov et al., 2007). They can be classified, according to their genomic organization relative to protein-coding transcripts (Rinn and Chang, 2012), as: (i) overlapping transcripts (sense or antisense, promoter-/intronic-/3^′^UTR-associated); (ii) divergent transcripts, which share the same promoter with coding genes, but are transcribed in the opposite direction; (iii) intergenic transcripts (lincRNAs), which are located in gene-desert regions (see **Figure 2**).

Genetic loci of lncRNAs are similar to those of mRNAs, sharing the same transcriptional machinery (Pol II), same layers of epigenetic regulation (such as histone-modification profiles) and splicing signals (Guttman et al., 2009; Derrien et al., 2012)*.* Almost half of the lncRNAs are also capped and polyadenylated. LncRNAs are frequently bi-exonic and localize predominantly in the nucleus. Their expression is highly cell-type specific, but on average to a lesser extent than protein-coding genes (Ravasi et al., 2006; Cabili et al., 2011; Djebali et al., 2012). Although most lncRNAs have very low translational potential, ribosome profiling (a technique that uses deep sequencing of ribosome-protected fragments to monitor *in vivo* translation) revealed that many lncRNAs can be engaged by ribosomes, and some have the potential of producing small peptides (<100 amino acids), whose biological significance is completely unknown (Ingolia et al., 2011; Bazzini et al., 2014).

Identification of lncRNAs is not a trivial task, but requires unbiased RNA detection methods, precise mapping in the genome within regions distinct from those occupied by coding transcripts, and analysis of protein-coding potential. Current approaches applied to lncRNA identification include: (i) tiling microarrays; (ii) unbiased RNA cloning techniques (SAGE and CAGE); (iii) massive parallel sequencing of transcripts (RNA-sequencing, RNA-seq); and (iv) chromatin immunoprecipitation combined with DNA sequencing (ChIP-seq). The latter approach is based on the identification of chromatin domains associated with active transcription, such as those with histone H3 lysine 4 trimethylation (H3K4me3) at the promoter, and histone H3 lysine 36 trimethylation (H3K36me3) on the transcribed gene body [known as “H3 K4-K63 domains,” (Mikkelsen et al., 2007; Guttman et al., 2009)]. Mapping in the genome is easier for lncRNAs that are located in gene-desert regions (intergenic – lincRNAs), while strand-specific reactions (strand-specific RT-qPCR or sequencing) are needed to identify overlapping lncRNAs. The coding potential of lncRNAs is typically assessed by searching for any open reading frame (ORF) that match known proteins or domains. However, this approach could miss small ORFs or newly evolved proteins. Alternatively, methods that use the codon substitution frequency to determine the likelihood that a sequence is protein coding can be exploited (Lin et al., 2011).

In contrast to miRNAs, which are highly conserved and mainly involved in negative regulation of gene expression at the post-transcriptional level, lncRNAs are poorly conserved and could regulate gene expression (either positively or negatively) at numerous levels by a variety of mechanisms (summarized in **Figure 2**), some of which are yet to be characterized (Cech and Steitz, 2014). Expression of lncRNAs occurs at a very precise time and/or developmental stage, suggesting a general role as “molecular signals” that integrate developmental cues or respond to different stimuli (Wang and Chang, 2011). Due to their purely transcriptional nature, lncRNAs could function immediately after transcription (with no need for protein translation) and act either locally, by affecting the expression of neighboring genes (*cis-acting*), such as during imprinting, or at distant sites (*trans-acting*; Ebisuya et al., 2008; Guttman and Rinn, 2012). Even in the absence of a regulatory function, the transcription of lncRNAs could serve as a signal *per se*, interfering or fostering the expression of neighboring/overlapping genes, as in the case of the lncRNAs, AIR, and XIST (Rinn and Chang, 2012).

The regulatory function of lncRNAs could also depend on their interaction with other molecules. In this case, lncRNAs act as “decoys” that titrate away transcription factors, splicing proteins or even miRNAs, thus relieving the activation/inhibition on target genes. Examples of such lncRNAs include TERRA, PANDA, PTENP, linc-MD1, and linc-ATB. Alternatively, lncRNAs could function as a “guide,” directing ribonucleoproteic complexes to specific loci to control gene expression locally (on neighboring genes) or at distant sites. Lastly, lncRNAs could function as scaffolds (e.g., HOTAIR – HOX antisense intergenic RNA), upon which other molecular components are assembled, bringing together independent functions/activities (Mercer et al., 2009; Ponting et al., 2009; Wang and Chang, 2011).

## NORMAL AND CANCER MAMMARY STEM CELLS

### THE STEM CELL COMPARTMENT IN THE NORMAL MAMMARY GLAND

**FIGURE 3:**
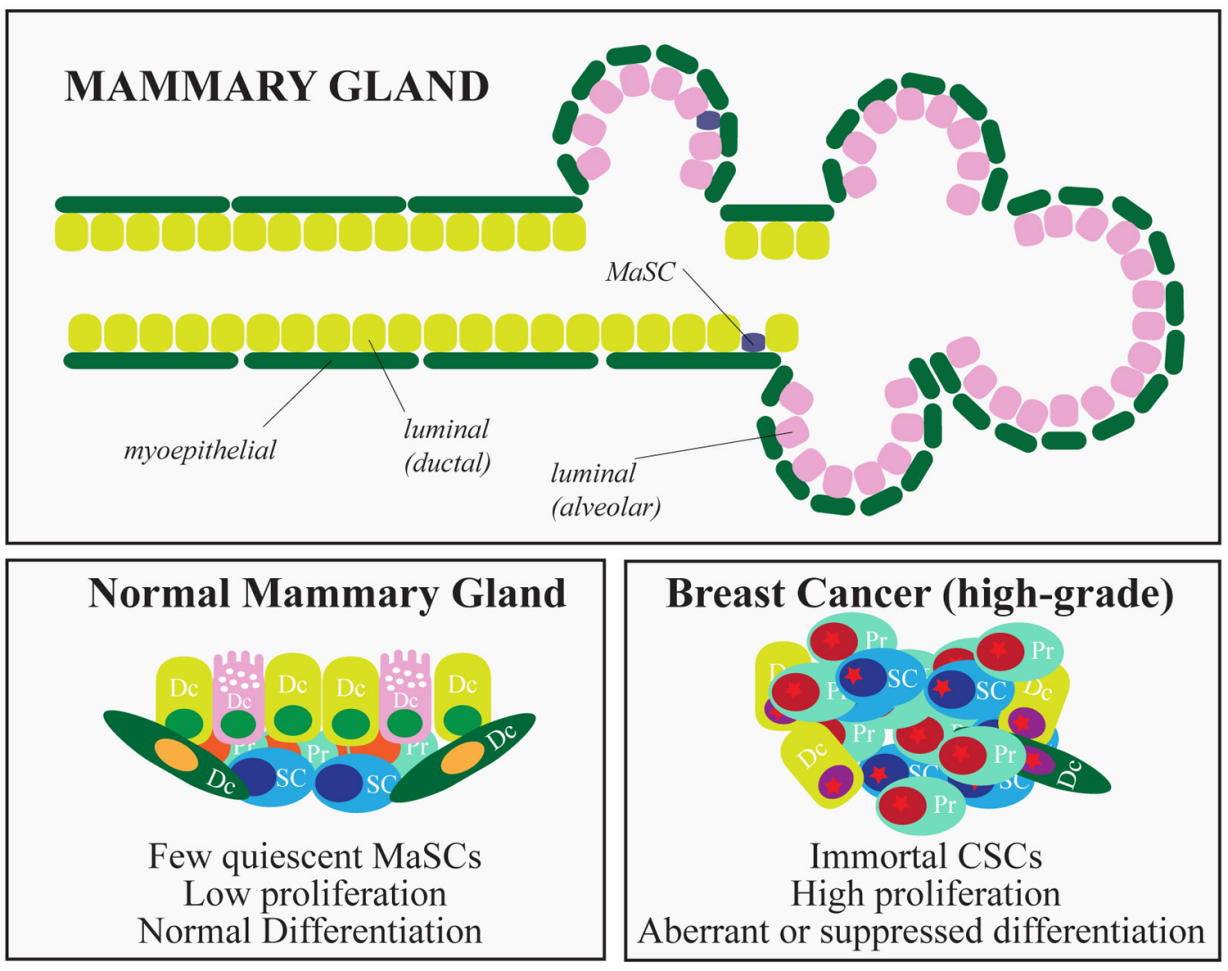
**Hierarchical organization of the mammary gland and breast cancer subtypes.** The figure depicts the epithelial components of the mammary gland. In the lower part, the characteristics of normal and cancer stem cells are summarized. (SC, stem cells; PC, progenitors; DC, differentiated cells).

The mammary gland is a glandular epithelium composed of milk-secreting hollow cavities, named *alveoli*, joined together by ducts to form groups termed *lobules.* Ducts also connect different lobules and eventually merge into the lactiferous duct that opens into the nipple. Mature cells that compose the mammary epithelium include luminal cells, in the inner layer of the mammary gland, and myoepithelial cells, located in the outer layer of the gland. The latter cells make contact with the basement membrane, physically sustaining the organ and providing the contractile force needed during lactation (**Figure 3**). The mammary gland is a highly dynamic organ that undergoes important morphogenetic changes during adult development (puberty) or during pregnancy-lactation and involution (Visvader and Stingl, 2014). Remarkably, the mammary gland maintains the ability to perform structural remodeling for several cycles, suggesting the existence of a reservoir of adult stem cells (SCs) able to sustain multiple rounds of pregnancy-lactation-involution and to generate all the cellular lineages that compose the gland.

Pioneering experiments in mice, using transplantation of entire sections of the gland (Deome et al., 1959) or isolated epithelial cells (Stingl et al., 2006), proved the existence of a multipotent population of rare cells able to reconstitute the entire organ: normal mammary stem cells (MaSCs). This *in vivo* reconstitution assay has become the gold standard in MaSC identification (Shackleton et al., 2006; Stingl et al., 2006; Cicalese et al., 2009) and has allowed the evaluation of mammary repopulating units (MRUs, functionally synonymous with MaSCs) in defined cellular subsets transplanted at limiting dilution, as a quantitative measure of MaSC abundance (Visvader, 2009). Lineage tracing experiments and reporter genes have helped to verify the clonality of mammary outgrowths, suggesting that unipotent and multipotent MaSCs coexist, endowed with the ability to repopulate the mammary gland by generating the different luminal and basal mammary lineages (Kordon and Smith, 1998; Van Keymeulen et al., 2011; Rios et al., 2014; Wang et al., 2014). Similarly to most adult SCs, MaSCs possess a number of defining characteristics: (i) multi-lineage differentiation potential (they can generate both luminal and basal lineages); (ii) ability to self-renew, usually through an asymmetric type of cell division, which generates one daughter SC and one progenitor, and maintains homeostasis of the SC pool; (iii) quiescence (or slow rate of division), unless activated, as occurs during pregnancy or lactation; and (iv) ability to withstand *anoikis*, surviving in anchorage-independent conditions.

An important advance in MaSC biology derived from the work of Dontu et al. (2003), who developed an *in vitro* methodology, the mammosphere assay, which maintains MaSCs in an undifferentiated condition and facilitates the study of self-renewal mechanisms. The assay exploits the ability of SCs to grow in anchorage independent conditions as clonal spheroids, composed of quiescent SCs, progenitors and somewhat differentiated cells. Under these culture conditions, MaSCs maintain their fundamental properties (self-renewal and multilineage differentiation potential) and can recapitulate mammary outgrowths when transplanted *in vivo* (Dontu et al., 2003).

### BREAST CANCER AND MAMMARY CANCER STEM CELLS

Breast cancer is the main disease of the mammary gland and one of the most common life-threatening diseases for women in Western countries (DeSantis et al., 2014). Thanks to the advent of genome-wide approaches, both oncologists and biologists have realized that breast cancer is a quite heterogeneous disease, and have attempted to classify tumors according to their molecular characteristics. This has led to a classification of breast tumors into at least five different molecular subtypes, based on their peculiar transcriptional profile: Luminal A, Luminal B, Her2-positive, Claudin-low, and Basal tumors (Perou et al., 2000; Sorlie et al., 2001; Sotiriou et al., 2003; Lehmann et al., 2011; Prat and Perou, 2011). These findings have revealed the molecular basis for breast tumor heterogeneity and suggested that different subtypes should be treated as different diseases, with tailored treatments and therapeutic strategies (Sotiriou and Pusztai, 2009).

As morphogenesis and homeostasis of adult tissues are sustained by SCs, it has been hypothesized that a similar mechanism might fuel the growth of tumors, relying on the existence of subpopulations of cancer cells with stem-like properties (cancer stem cells – CSCs, Medema, 2013). The CSC hypothesis implies that tumors are hierarchically organized like normal tissues, with a subset of tumor cells at the top of the hierarchy possessing the ability to self-renew and to differentiate, albeit aberrantly. Breast cancer was one of the first solid malignancies in which CSCs were identified and characterized, mostly immunophenotypically as lin^-^/CD44^+^/CD24^-^ cells (Al-Hajj, 2003). Mammary CSCs were the only cells able to sustain tumor growth in NOD/SCID mice, whereas cells that did not express the CSC markers were non-tumorigenic (Al-Hajj, 2003). Mammary CSCs are also considered to be responsible for relapse and metastasis. This contention is based on multiple observations: (i) poorly differentiated, more aggressive breast cancers tend to be CSC-rich, compared to more highly differentiated, less aggressive, CSC-poor breast tumors (Pece et al., 2010); (ii) mammary CSCs are relatively resistant to both radiation treatments and cytotoxic chemotherapy *in vitro* and *in vivo* (Liu and Wicha, 2010), a property reminiscent of the intrinsic ability of MaSCs to withstand genotoxic stress; and, finally, (iii) the proportion of cells with CSC properties is typically increased after conventional therapies (Li et al., 2008; Creighton et al., 2009).

Recent research has provided evidence of functional plasticity within the SC compartment, suggesting that CSCs should not be considered as a fixed entity, solely derived from the transformation of a normal SC, but rather as the result of the acquisition of “stemness” properties by tumor cells (Gupta et al., 2011). In particular, in cancer cell populations’ bidirectional interconversion between CSCs and non-CSCs occurs under certain conditions (Roesch et al., 2010; Chaffer et al., 2011; Gupta et al., 2011). This plasticity is not a universal property of cancer cells and it is appears to be associated with certain tumor subtypes, such as breast basal carcinomas. Although it is currently unclear how frequently the interconversion between CSCs and non-CSCs occurs *in vivo*, cell state dynamics are dependent on external (i.e., microenvironment) and internal (i.e., genetic) cues, and rely on the same epigenetic, transcriptional and post-transcriptional mechanisms that control “stemness” in normal SCs (Roesch et al., 2010; Chaffer et al., 2011; Gupta et al., 2011).

One such mechanism is the epithelial-to-mesenchymal transition (EMT), a reversible transcriptional program that is physiologically activated during embryogenesis, allowing partial or complete transition of cells from an epithelial to a mesenchymal state (Thiery et al., 2009). Pathways leading to EMT are activated by specific stimuli, such as transforming growth factor beta (TGF-β) or fibroblast growth factor (FGF), through the stimulation of EMT transcription factors (EMT-TFs), such as members of the snail family (SNAIL1/2), bHLH family (TWIST), and ZFH family (ZEB1 and ZEB2), which repress epithelial gene expression and foster the establishment of a motile and invasive mesenchymal phenotype (Thiery and Sleeman, 2006). EMT program(s) have been frequently associated with cancer progression, metastasis and acquisition of SC-traits (Thiery et al., 2009; Hanahan and Weinberg, 2011). In breast cells, the expression of EMT-TFs, such as TWIST and SNAIL, induces mesenchymal and stem-related markers, increases mammosphere formation, expands the CSC population (CD44^+^/CD24^-^) and induces tumorigenesis (Mani et al., 2008).

Of note, both mouse (CD49f^high^/CD24^med^) and human MaSCs (CD44^high^/CD24^low^) express markers associated with EMT, such as N-cadherin, Vimentin, SNAIL1/2, and SLUG (Mani et al., 2008). In mouse models, the expression of the EMT–TF SLUG is sufficient to reprogram luminal progenitor cells (CD61^+^) to fully functional MaSCs suggesting that a certain degree of plasticity between SC and progenitor states also exists in the normal breast epithelium (Guo et al., 2012). Indeed, interconversion from an epithelial to a mesenchymal/stem-like state has been also observed in human mammary epithelial cells (Chaffer et al., 2011, 2013). In this context, the maintenance of self-renewal properties and protection from spontaneous differentiation is achieved through autocrine and paracrine signals, which involves TGF-β canonical and non-canonical Wnt pathway activation and EMT-TFs (SLUG, TWIST, ZEB1/2; Scheel et al., 2011).

Finally, and of relevance to the subject of this review, several ncRNAs have been associated with EMT in the breast gland, frequently acting together, in concert with chromatin regulators (CRs) and TFs. Relevant examples are miRNAs of the miR-200 family, miR-205, miR-7, miR-22, and some lncRNAs, including HOTAIR, linc-RoR, H19, and lncRNA-ATB (discussed below).

## CELL FATE SPECIFICATION BY miRNAs AND lncRNAs OCCURS BY INTEGRATING MULTIPLE SIGNALING PATHWAYS INVOLVED IN SC AND CSC BIOLOGY

**FIGURE 4:**
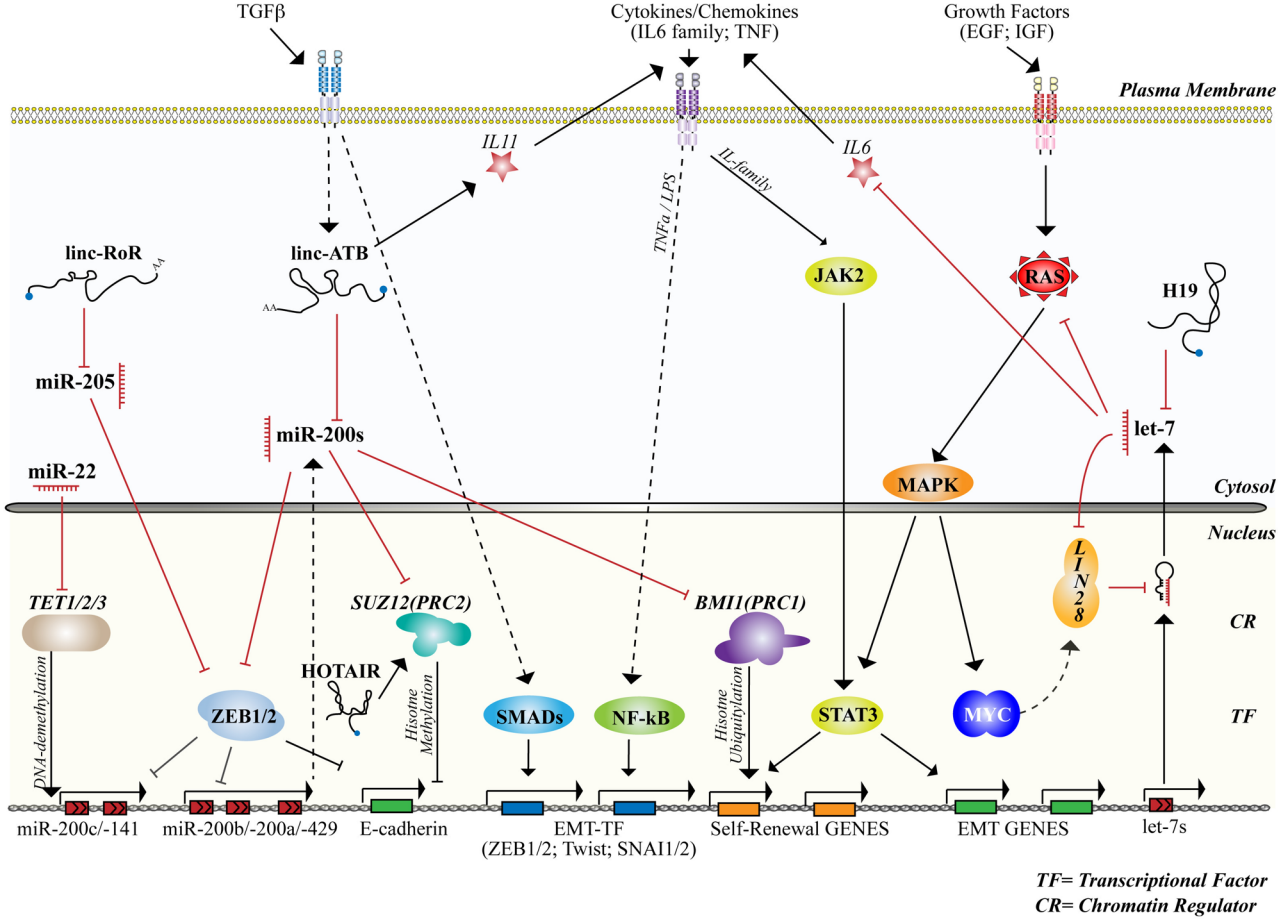
**microRNAs and lncRNAs control stemness and differentiation by interacting with signaling and transcriptional/epigenetic networks.** The figure summarizes the activity of the main miRNAs and lncRNAs involved in the control of normal or cancer mammary SCs together with signaling networks (cytosol) and the transcriptional/epigenetic framework (nucleus) to which they belong or that they regulate. Straight and dashed arrows refer to direct or indirect interaction/regulation, respectively. Red lines mark inhibitory interactions. The activity of transcriptional factors (TFs) and chromatin regulators (CRs), cited in the text and involved in the control of stemness and differentiation, are also shown.

As previously mentioned, the activity of ncRNAs, either miRNA or lncRNAs, is integrated with signaling networks and the transcriptional framework, thus, generating complex circuits that control cell fate and differentiation. Hereafter, we will discuss the current knowledge of the involvement of ncRNAs in the biology of MaSCs and of mammary CSCs (summarized in **Tables 1** and **2**, and schematized in **Figure 4**).

**Table 1:** List of microRNAs (miRNAs) and long ncRNAs (lncRNAs) associated with normal breast stem cells (SCs).

ncRNAs	Cell Type	Function	Signaling upstream	Signaling downstream	Target genes	Reference
Let-7	Normal breast cells (MCF10A) Human	Inhibition of self-renewal/promoter of differentiation	SRC/IL-6/NF-kB	LIN28/Let-7/STAT3		Iliopoulos et al. (2009)
Let-7/miR-205/miR-22	Mammary gland progenitors (Comma-Dβ) Mouse	Inhibition of self-renewal by let-7				Ibarra et al. (2007)
miR-205	Breast epithelial cells (HMEC) Human	Inhibition of EMT	JAG1/NOTCH2	Hes1/ZEB1	ZEB1/NOTCH2	Chao et al. (2014)
miR-200c	Human primary breast SCs (CD44^+^/CD24^-^)	Inhibition of self-renewal			BMI/PRC1	Shimono et al. (2009)
miR-200c	Human normal breast cell lines (MCF12A – HMEC)	Inhibition of EMT/stemness	p53		ZEB1/BMI-1	Chang et al. (2011)
miR-22	Normal breast cells (MCF10A – HMEC) and Mouse models	Promoter of EMT and stemness		ZEB1–ZEB2	TET and miR-200 inhibition	Song et al. (2013)
miR-205/-200 family	MDCK cells	Maintenance of the epithelial state	TGF- β		ZEB1–ZEB2	Gregory et al. (2008)

*The table summarizes the miRNAs and the lncRNAs linked to stem cells or progenitors of the mammary gland, cited in the text. Shown are: the cell type or tumor type (source) in which the miRNA/lncRNA has been investigated, the function in that compartment, the upstream and downstream signaling, the target genes, and the appropriate references.*

**Table 2:** List of miRNAs and lncRNAs associated with breast cancer stem cells (CSCs).

ncRNAs	Source	Function	Signaling upstream	Signaling downstream	Target genes	Reference
Let-7	lin/CD44^+^/CD24^-^ (SK-3^rd^) Human	Inhibition of self-renewal			H-RAS and HMGA2	Yu et al. (2007)
Let-7	Human breast cells transformed with Src (MCF10A ER-SRC)	Inhibition of cell transformation	SRC/IL-6/NF-kB	LIN28/Let-7/STAT3		Iliopoulos et al. (2009)
Let-7	TN and HER2 breast cancers Human	Inhibition of CSC maintenance	SHP2	MAPK/ERK and MYC	RAS/MYC	Aceto et al. (2012)
Let-7/miR-200 families	Breast cancer cells (MCF7) Human	Inhibition of EMT	JAK2/STAT3	LIN28/Let-7 and miR-200/ZEB1	HMGA2/ZEB1	Guo et al. (2013)
miR-205/-200 family	Human primary breast cancers/ MDCK cells	Maintenance of the epithelial state/tumor suppression	TGF -β		ZEB1–ZEB2	Gregory et al. (2008)
miR-205	Breast cancer cells MDA-MB-231 – BT459 – PT Human	Inhibition of EMT and mammary tumorigenesis	JAG1/NOTCH2	Hes1/ZEB1	ZEB1/NOTCH2	Chao et al. (2014)
miR-200c	Human primary BCSCs (CD44+/CD24^-^)	Inhibition of self-renewal			BMI/PRC1	Shimono et al. (2009)
miR-200b	Human CD44^+^/CD24^-^ from MCF10A-SRC	Inhibition of self-renewal/invasion and tumor growth			SUZ12/PRC2 and E-cadherin	Iliopoulos et al. (2010)
miR-200b/-200c	Multiple human breast cancer cells	Interconversion of CSCs to non-CSCs	TGF -β		ZEB1	Chaffer et al. (2013)
miR-200c/-141	Human cancer cells (MDA-MB-231)	Inhibition of EMT/stemness/survival			Jag1/Maml2/3/ZEB1	Brabletz et al. (2011)
miR-200c	Human breast cancer cell lines BT549	Inhibition of EMT/stemness	p53		ZEB1/BMI-1	Chang et al. (2011)
miR-22	Human breast cancer and Mouse models	Promoter of metastasis, EMT, invasiveness and stemness		ZEB1–ZEB2	TET and miR-200 inhibition	Song et al. (2013)
HOTAIR	Human breast cancer cells (MCF -7; MCF-10A; SK-BR3; MDA-MB-231)	Promoter of tumor metastasis and invasion		Metastasis suppressor genes	PRC2 and chromatin state of several genes	Gupta et al. (2010)
HOTAIR/miR-7	Breast cancer cell lines (MCF -7; MDA-MB-231)	Promoter/Inhibitor (HOTAIR/miR-7) of EMT/stemness		STAT3	miR-7:SETDB1 HOTAIR:HoxD10	Zhang et al. (2014).
Linc-ROR	iPSCs/ES cells	Maintenance of self-renewal	Sox2, Nanog, Oct4	Self-renewal genes	Sponge for miR-145 family	Wang et al. (2013b)
Linc-ROR	Multiple human breast cancer cells	Promoter of EMT/stem-like features			Sponge for miR-205	Hou et al. (2014)
Lnc-H19	Muscle cells and cancer cells	Promoter of muscle differentiation		Differentiation genes/let-7 targets	Sponge for let-7 family	Kallen et al. (2013)
Lnc-H19/miR-675	Human breast cancer and cell lines (MDA-MB-468)	EMT/promoter of tumor metastasis	TGF -β hypoxia/HGF /SF	PI3K/AKT/Slug	E-cadherin	Matouk et al. (2007)
Lnc-ATB	Hepatocellular carcinomas and Human Breast cancer cells (MCF-7)	EMT/promoter of tumor metastasis	TGF-β/IL-11	ZEB1–ZEB2/STAT3	Sponge for miR-200 family	Yuan et al. (2014)

*The table summarizes the miRNAs and the lncRNAs, linked to breast cancer stem cells, cited in the text. Shown are: the cell type or tumor type (source) in which the lncRNA has been investigated, the function in that compartment, the upstream and downstream signaling, the target genes and the appropriate references.*

### LET-7 FAMILY AND IL6/STAT3 CIRCUITRY

The first report showing the involvement of a miRNA in mammary CSCs was in 2007, when Yu et al. (2007) described their observation that let-7 levels were markedly reduced in tumor-initiating cells (TICs, defined as lin^-^/CD44^+^/CD24^-^, and operationally equal to CSCs) compared to their non tumorigenic counterparts. To identify miRNAs potentially involved in the control of CSC biology, they used a derivative of the SK-BR-3 breast cancer cell line, named SK-3rd, obtained by serial passaging of the original cell line in NOD/SCID mice treated with chemotherapy. As chemotherapy typically increases the proportion of CSCs within breast tumors, the SK-3rd derivative had an increased proportion of CSCs compared with the parental line, as assessed by the mammosphere assay, cell surface marker expression (CD44^+^/CD24^-^) and *in vivo* xenotransplantation assays. Several members of the let-7 family (plus other miRNAs, such as the miR-200 family) were depleted in SK-3rd cells as compared to the parental cells. Importantly, the same miRNAs were downregulated in CSCs from clinical breast cancer samples (lin^-^/CD44^+^/CD24^-^) compared to their non-tumorigenic counterparts. The overexpression of let-7 in mammary CSCs caused a striking impairment in proliferation, mammosphere-forming ability, and tumor formation and metastasis *in vivo*. Mechanistically, the levels of let-7 inversely correlated with those of two of its direct targets, H-RAS and HMGA2, which are involved in SC self-renewal and multipotency regulation. Silencing of these two targets in mammary CSCs partially recapitulated the effects of let-7 overexpression, suggesting that the repression of ‘stemness’ traits by let-7 is, in part, mediated by the repression of H-RAS and HMGA2 (Yu et al., 2007).

In the mouse, members of the let-7 family were shown to be expressed at low levels in self-renewing progenitors (ALDH^+^/Sca-1^+^) and induced upon differentiation, suggesting that low levels of let-7 mark the self-renewal compartment and could be used to prospectively isolate MaSCs (Ibarra et al., 2007). Accordingly, the let-7 family emerged as the most induced group of miRNAs upon estradiol treatment in human luminal cells (MCF-7; Bhat-Nakshatri et al., 2009).

Several independent studies further suggested that miRNAs of this family are implicated in the self-renewal of CSCs in breast and other cancers, by multiple mechanisms. One of these mechanisms involves the pluripotency gene known as LIN28/LIN28B, which inhibits the function of let-7 by interfering with its biogenesis (Piskounova et al., 2011). LIN28 is frequently overexpressed and associated with advanced malignancy in multiple cancer types (Viswanathan et al., 2009). In breast cancer, LIN28 expression confers CSC-traits and impinges on signaling mechanisms involved in self-renewal of normal and cancer SCs, such as the Wnt/beta-catenin pathway, NF-kB signaling, and inflammatory cytokine signaling (Iliopoulos et al., 2009; Cai et al., 2013). Members of the interleukin-6 (IL-6) family, which are induced in a paracrine/autocrine fashion in breast cancer, mediate the activation of the Janus Kinase 2 (JAK2) and of the signal-transducer and transcription factor 3 (STAT3) promoting the expansion of mammary CSCs (Marotta et al., 2011; Yu et al., 2014). The IL-6/STAT3 axis can induce LIN28 expression, which in turn inhibits let-7 activity, thus generating a positive feedback loop (let-7 targets IL-6) that confers SC traits to mammary epithelial cells (Iliopoulos et al., 2009; Guo et al., 2013).

A similar loop is also operational downstream of the Src-homology 2 domain-containing phosphatase SHP2: a protein-tyrosine phosphatase encoded by the PTPN11 locus. SHP2 is a transducer of RTK and cytokine-receptor signaling that promotes breast cancer progression and a CSC-phenotype in ER-negative cancers (Aceto et al., 2012). In this case, a signaling cascade that involves the mitogen-activated protein kinase (MAPK)/extracellular signal-related kinase (ERK) pathway and the MYC-oncogene is activated by SHP2 and sustained through let-7 downregulation. Indeed, let-7 targets multiple genes, including RAS and MYC, which feedback on ERKs and LIN28, respectively, thus creating a loop that maintains CSCs and fosters metastasis in HER2+ and triple-negative breast cancers (Aceto et al., 2012).

### THE H19 lncRNA AND BREAST CANCER METASTASIS

Human H19 was the first lncRNA with no coding potential to be described, opening the door to the so-called “non-coding revolution” (Brannan et al., 1990). The H19 gene belongs to a conserved imprinted region on human chromosome 11, located near the insulin-like growth factor 2 (*IGF2*) gene, and encodes a 2.3 kb long, cytoplasmic, capped, and polyadenylated ncRNA that functions primarily in the epigenetic silencing of the *IGF2* gene (Gabory et al., 2010). H19 is strongly induced during embryogenesis and selectively expressed by the maternally inherited chromosome, with the function of silencing *in cis* the maternal *IGF2* allele, thereby allowing selective expression of the paternal allele (Bartolomei et al., 1991; Gabory et al., 2010).

In adult tissues, H19 expression is retained in muscles (skeletal muscles and the heart), or is suddenly activated in cancers, where it is believed to act mainly as an oncogene (Matouk et al., 2007; Gabory et al., 2010). H19 functions in the adult are linked to miRNA circuits: H19 RNA is the precursor of miR-675, which is encoded in its first exon and involved in muscle development and regulation of EMT (Cai and Cullen, 2007; Matouk et al., 2014). Furthermore, H19 acts as a “decoy” for miRNAs of the let-7 family during muscle differentiation (Kallen et al., 2013). By this mechanism, the expression of H19 relieves the repression on endogenous let-7 targets, such as HMGA2 and DICER, potentially contributing to the onset of the transformed phenotype (Kallen et al., 2013). Indeed, H19 expression is high in some cancers, including breast carcinomas, where it is associated with metastasis and the acquisition of EMT traits (Matouk et al., 2014). Several mechanisms can induce H19 expression in breast cancer cells, including: (i) activation from within the cell by upregulation of E2F1 (Berteaux et al., 2005), c-MYC (Barsyte-Lovejoy et al., 2006), or the loss of the tumor suppressor p53 (Dugimont et al., 1998); or (ii) activation by external stimuli, such as TGF-β, hypoxia, and HGF/SF. In this context, high levels of H19 could promote the loss of E-cadherin and the upregulation of the EMT-inducer SLUG to reinforce the mesenchymal state, through as yet an unknown mechanism that implicates both miR-675 and let-7 (Matouk et al., 2014).

### THE miR-200 FAMILY IN BETWEEN EMT AND CHROMATIN REGULATION

In human primary samples, Shimono et al. (2009) identified a signature of 37 miRNAs differentially expressed between mammary CSCs and their non-tumorigenic counterparts. In particular, several members of the miR-200 family were found to be downregulated in mammary CSCs. This family comprises five members, which are organized in two conserved genomic clusters, one containing miR-200b, -200a, and -429 and the other encompassing miR-200c and miR-141 (**Figure 4**). miRNAs of this family were also found to be downregulated in MaSCs isolated by flow cytometry (lin^-^/CD49f^high^/CD24^med^/CD29^high^) from the normal mouse mammary gland. Forced expression of one member of the family, miR-200c, repressed both normal mammary outgrowths in mammary gland reconstitution assays, and the *in vivo* tumorigenicity of human and mouse mammary CSCs. Mechanistically, this was linked to the ability of miR-200c to target BMI1 (B-lymphoma Mo-MLV insertion region 1 homolog), a component of the Polycomb Repressing Complex 1 (PRC1) and a critical regulator of SC self-renewal and differentiation (Shimono et al., 2009).

In a different setting, members of the miR-200 family were found to be downregulated in mammary CSCs (CD44^high^/CD24^low^) isolated from MCF10A cells transformed with the SRC oncogene (Iliopoulos et al., 2010). In this context, miR-200(s) targets SUZ12, a component of the PRC2, which epigenetically controls the expression of several genes, including E-cadherin, by H3K27 trimethylation. The miR-200/SUZ12/E-cadherin axis appeared to be important for the maintenance of mammary CSCs and in the regulation of metastasis. Overexpression of miR-200b or loss of SUZ12 expression inhibited mammosphere formation, invasion, and tumor growth from genetically distinct breast cancer cell lines, and cooperated with chemotherapy to prevent tumor relapse in xenograft models (Iliopoulos et al., 2010).

The notion of regulation of CSC traits by miR-200 has been further confirmed in multiple models (Brabletz et al., 2011; Chang et al., 2011; Chaffer et al., 2013; Lim et al., 2013; Song et al., 2013), and tightly linked to the function of EMT–TFs of the ZEB1/2 and to EMT (Christoffersen et al., 2007; Hurteau et al., 2007; Burk et al., 2008; Gregory et al., 2008; Korpal et al., 2008; Park et al., 2008; Scheel et al., 2011). Indeed, the expression of miR-200 family members is highly enriched in epithelial cells, almost absent in mesenchymal or basal cells, and positively correlates with the epithelial marker E-cadherin (Park et al., 2008). The E-box binding factors ZEB1 and ZEB2 possess a remarkable number of conserved binding sites for miR-200 miRNAs. A total of eight MREs are present in the 3^′^UTR of ZEB1 (5 for miR-200bc/429, and 3 for miR-200a and -141) and nine in ZEB2 (six for miR-200bc and three more for miR-200a/-141) (Christoffersen et al., 2007; **Figure 4**). Conversely, miR-200 genetic loci possess multiple binding site for ZEB1/2, generating a double negative feedback loop that controls the epithelial or mesenchymal phenotype (Burk et al., 2008; Gregory et al., 2008; Korpal et al., 2008). This “switch” mechanism is one of the most representative examples of a transcriptional circuitry that regulates cell fate. The two players, ZEB1/2 and miR-200, which regulate the mesenchymal and the epithelial fate, respectively, are never in equilibrium: as one of the two starts accumulating the other disappears, critically contributing to the establishment of cell fate.

Studies from the Weinberg’s lab, suggested a role for the ZEB1/miR-200 axis also in the regulation of cancer cell plasticity, by integrating external stimuli from the microenvironment (such as TGF-β) with epigenetic mechanisms (Chaffer et al., 2013). In several breast cancer cell lines, CD44^low^ (non-CSC) and CD44^high^ (CSCs) cells can interconvert into each other by regulating ZEB1 expression and, thus, miR-200 levels. Mechanistically, this occurs through the epigenetic regulation of the ZEB1 promoter, which converts from a poised state (with the coexistence of active and inactive chromatin marks – such as H3K4me3 and H3K27me3) to an active state (by removal of the repressing mark – H3K27me3) in response to TGF-β signaling (Chaffer et al., 2013).

Finally, the ZEB1/miR-200 axis has been shown to interact with other pathways associated with SC-biology, such as the Notch pathway, by interfering with the ligand Jagged-1 (JAG1) and the mastermind-like co-activators MAML2 and MAML3 (Brabletz et al., 2011). Besides ZEB1 activity, at least two other mechanisms have been described that regulate miR-200 expression in mammary cells. The tumor suppressor p53 can regulate the expression of miR-200c, and the acquisition of EMT and SC properties, by directly binding and transactivating the miR-200c/-141 locus in human normal mammary epithelial cells (Chang et al., 2011). Alternatively, the regulation of miR-200 clusters can be achieved epigenetically, by H3K27me3 histone modification at the miR-200b-a-429 locus (associated with gene silencing) induced upon autocrine TGF-β signaling (Lim et al., 2013), or by CpG hypermethylation of promoter regions (Gregory et al., 2011).

### lncRNA-ATB AND EMT UPON TGF-β STIMULATION

The EMT program activated by TGF-β stimulation also involves a lncRNA, named lncRNA–ATB (Activated by TGF-β). This cytoplasmic lncRNA has been identified among a group of 100s of ncRNAs that are regulated upon TGF-β stimulation in hepatocellular carcinomas, breast and colorectal cancer cell lines (Yuan et al., 2014). Bioinformatics predicted that lncRNA–ATB could act as a competing endogenous RNA for the miR-200 family, due to three putative binding regions for miR-200 (Yuan et al., 2014). Indeed, when expressed in cells, lncRNA–ATB phenocopies the pro-metastatic role of TGF-β by inducing EMT and metastatic colonization. Mechanistically, this occurs through the positive modulation of the miR-200 targets, ZEB1 and ZEB2, via competition of lncRNA–ATB over miRNA-200(s). In hepatocellular carcinomas, high levels of lncRNA–ATB are predictive of poor survival and distant metastasis. Of note, while the EMT induction by lncRNA–ATB, and the early phase of tumor dissemination, could be fully rescued by miR-200 overexpression, the late metastatic colonization of lung and liver is miR-200-independent and mediated by the stabilization of IL-11 mRNA (directly bound by lncRNA–ATB), which in turn activates STAT3 signaling (Yuan et al., 2014). Hence, lncRNA–ATB drives EMT and metastasis by two independent mechanisms that impinge on different signaling modules that control CSC-traits (ZEB/miR-200s and IL-11/STAT3).

### miR-22 AND TET-FAMILY DEPENDENT CHROMATIN REMODELING

miR-22 provides yet another regulatory module of miR-200 levels in mammary cells. This miRNA acts as an epigenetic regulator of EMT, stemness, and metastasis in estrogen receptor positive (ER+) breast cancers through the regulation of hypermethylation of the miR-200 promoter (Song et al., 2013). miR-22, which has been also found to be highly expressed in murine progenitor mammary cells (Ibarra et al., 2007), is upregulated in non-triple-negative breast cancers, positively correlating with high tumor grade and poor clinical outcome (Buffa et al., 2011; Enerly et al., 2011; Gregory et al., 2011; Song et al., 2013). When overexpressed in human or mouse mammary cells, miR-22 induces a transcriptional reprogramming, with upregulation of ZEB1/2 and downregulation of the miR-200 family, resulting in a mesenchymal phenotype, expansion of the MaSC pool, tumorigenesis and metastasis (Song et al., 2013). Mechanistically, these phenotypes are dependent on the methylation status of the miR-200 promoter and on the regulation of the TET (10 eleven translocation) family enzymes. These enzymes (TET1, TET2, and TET3) can modify DNA by removing the repressive 5-methylcytosine mark (5mC) by hydroxylation and are all targeted by miR-22 (Tahiliani et al., 2009; Ito et al., 2010).

### THE miR-205/ZEB CIRCUIT AND linc-RoR

miR-205 was identified as the most abundantly expressed miRNA in progenitor cells of the mammary gland, purified using ALDH as marker (Ibarra et al., 2007), suggesting a role for this miRNA in lineage specification and mammary gland formation. miR-205 is frequently downregulated in breast cancers and is associated with tumor relapse in patients with the triple negative subtype, which typically possess high CSC content (Sempere et al., 2007; Wang et al., 2013c). miR-205 is involved in the regulation of EMT through the targeting of ZEB1 (one conserved site) and ZEB2 (two conserved sites) and acts cooperatively with miRNAs of the miR-200 family in the specification of epithelial vs. mesenchymal fate (Gregory et al., 2008). A novel circuitry, which integrates signaling from the microenvironment with the induction of EMT has recently been described in breast cancer (Chao et al., 2014). In this case, the repression of miR-205 is achieved by the activation of Notch, mediated by the release of the ligand JAG1 by the tumor stroma, and the binding of the HES1 transcriptional repressor (an effector of the Notch pathway) directly to the miR-205 promoter. Loss of miR-205 increases ZEB1/2 levels and those of another target, NOTCH2, generating a feedback loop that controls epithelial polarization (by LLGL1 and LLGL2 genes), symmetric cell division, EMT, and mammary tumorigenesis (Chao et al., 2014).

This complex circuitry also involves the activity of at least another regulatory RNA, linc-RoR, a lncRNA involved in pluripotency and embryonic SCs (Loewer et al., 2010). Linc-RoR was found to be upregulated in breast tumor samples and was able to induce EMT in human immortalized mammary epithelial cells (MCF10A). The function of linc-RoR in the maintenance of induced pluripotent stem cells (iPSCs) and self-renewal of embryonic stem cells (ES cells) has been linked to its cytosolic localization and its ability to function as a competing endogenous RNA for tumor suppressive miRNAs (such as miR-145; Wang et al., 2013b). Similarly, in the breast compartment, linc-RoR interacts with endogenous miR-205 and induces the derepression of ZEB2, fostering the EMT program and the acquisition of SC traits by cancer cells (Hou et al., 2014).

### HOTAIR AND PRC2

HOX antisense intergenic RNA is a lncRNA generated by antisense transcription of the *HOXC* gene cluster (Rinn et al., 2007). The primary function of HOTAIR is during embryonic development, when it regulates the silencing of the distant *HOXD* locus (transregulation), acting as a scaffold and bringing two different chromatin remodeling complexes to the same locus to enforce gene silencing (Rinn et al., 2007). This recruitment of chromatin remodelers is mediated by two different RNA domains in HOTAIR: (i) one comprising the first 300 nts of HOTAIR, which physically associate with the PRC2 complex via EZH2 to induce H3K27me3; and (ii) the other comprising the last 700 nts of HOTAIR, which bind to the LSD1-coREST complex that demethylates H3K4me2, hence reinforcing transcriptional repression (Tsai et al., 2010).

HOX antisense intergenic RNA is also frequently upregulated in breast cancer metastasis, and its expression in primary tumors is a predictor of invasiveness and adverse outcome, particularly in ER-positive cancers (Gupta et al., 2010; Gutschner and Diederichs, 2012). The overexpression of HOTAIR is sufficient to promote invasion and metastasis of breast cancer cells by re-directing the activity of the PRC2 complex to hundreds of genes, including many metastasis suppressor genes (Gupta et al., 2010). Accordingly, HOTAIR silencing inhibits metastasis, especially in cells with high PRC2 activity (Gupta et al., 2010). Intriguingly, the Breast Cancer 1, early onset gene (*BRCA1*), whose mutations are responsible for a number of inherited breast cancers, inhibits the interaction between PRC2 and HOTAIR by competing with HOTAIR for binding to EZH2. This observation suggests that *BRCA1* loss could induce tumorigenesis also by an EZH2/HOTAIR-dependent mechanism (Wang et al., 2013a).

Elevated levels of HOTAIR are predictive of unfavorable prognosis in other cancers (such as colon and liver) pointing to a more general role in oncogenesis (Kogo et al., 2011; Yang et al., 2011). Indeed, EMT induced by TGF-β treatment in colon (DLD-1/HT-29) and mammary (MCF10A) cell lines was shown to involve and be dependent on HOTAIR expression (Pádua Alves et al., 2013).

Finally, since HOTAIR regulates the *HOXD* locus, it has been suggested that this lncRNA downregulates another miRNA associated with EMT, miR-7, which is dependent on *HoxD10* (Reddy et al., 2008). Indeed, miR-7 modulates the histone methyl transferase SETDB1, regulating a STAT3-dependent EMT, and acquisition of SC traits in CD44^+^ cells derived from MDA-MB-231 human breast cancer cells (Zhang et al., 2014).

## CONCLUDING REMARKS

By sitting at the intersection of complex circuitries that integrate transcriptional, post-transcriptional, and epigenetic control, ncRNAs exert a pervasive function on cell regulation. We are only starting to appreciate the relevance of this new layer of organization in the cellular ‘master plan,’ and the impact of its subversion in diseases. Indeed, deciphering the role of ncRNAs in cancer is especially desirable, not only from the perspective of understanding the molecular basis of this disease, but also with the view of developing novel clinical tools and treatments. With this outlook in mind, a number of important cultural and technological challenges lay ahead of us.

First, a true understanding of the functions of ncRNAs can be obtained only at the systems level. This seems to be necessitated by the very nature of the workings of this class of regulators, which act on multiple targets and are integrated in multiple cellular molecular functions, thus, affecting phenotypes in complex ways. Simply put, the reductionistic approach (even when sophisticated) that has served us reasonably well in deciphering gene function is bound to produce limited knowledge on the biology of ncRNAs. We will have to devise strategies that contemplate the integration of multiple omics approaches, including transcriptomics, proteomics, and epigenomics, in addition to phenotypical analysis, to understand the impact of the experimental modulation of the levels of ncRNAs. And yet, high throughput analysis will not suffice. High-resolution studies will also be needed, including quantitative assessment, analysis of threshold levels of action, resolution of the signals in space and time, dependency on cellular context and developmental stage.

In the particular case of lncRNAs, a series of additional limitations will have to be overcome. A major challenge will be to obtain the complete annotation of tissue-specific and/or cell type-specific lncRNAs, a task that might require years. More importantly, we will need to define genetic models to study their functions. This represents a formidable task: the genomic loci of lncRNAs are frequently located in gene-rich areas, often overlapping with other genetic units, which renders traditional knock-out or knock-in strategies less than ideal. Moreover, the fact that lncRNAs frequently exert positional effects *in cis* further complicates their analysis.

However, the field of applications for ncRNAs, especially in cancer, harbors great promise. The expression of ncRNAs, especially lncRNAs, is tightly and specifically regulated in time and as a function of the cellular context. This property makes them appealing diagnostic markers, since they can distinguish between cell types in the same tissue and between different functional states of the same lineage (for instance, cells poised for EMT). Their application as markers, for diagnostic/prognostic assessment and for therapy stratification seems, therefore, a reasonable perspective.

The ultimate goal, obviously, would be to exploit ncRNAs as therapeutic tools. The evidence, herein reviewed, that these molecules have a key role in cell fate determination and in the modulation of cellular plasticity, leading to the acquisition of stem-related properties, support such a possibility. One important advantage of ncRNA-based therapies would be the wide range of intracellular targets, which should minimize the possibility of escape (drug resistance) due to the acquisition of secondary mutations by cancer cells. One of the major hurdles, on the other hand, resides in the lack of suitable (and specific) vectors for delivery, a problem shared with all forms of gene therapy in cancer. However, ncRNAs might possess an intrinsic and exploitable advantage. Given their multi-target characteristics, the action of ncRNAs might be, at least in some cases, cell-context dependent. In other words, the modulation of ncRNA levels might not lead to stereotypical consequences in all cell types, but to (more or less) specific ones, as a function of the cell type. If so, the need for specific delivery might be alleviated, at least in part, if the “right” ncRNA can be selected to exert the desired effect only in the desired cell types. Clearly, a deeper understanding of the biological effects of the various ncRNAs will need to be acquired before this possibility can be actualized into real therapeutic strategies.

## Conflict of Interest Statement

The authors declare that the research was conducted in the absence of any commercial or financial relationships that could be construed as a potential conflict of interest.
